# μ-Oxido-bis­({2,2′-[*o*-phenylenebis(nitrilo­methylidyne)]diphenolato}iron(III)) methanol monosolvate dihydrate

**DOI:** 10.1107/S1600536810031533

**Published:** 2010-08-11

**Authors:** Jia-Hao Yan, Xiao-Ping Shen, Hu Zhou

**Affiliations:** a School of Chemistry and Chemical Engineering, Jiangsu University, Zhenjiang 212013, People’s Republic of China; b School of Materials Science and Engineering, Jiangsu University of Science and Technology, Zhenjiang 212003, People’s Republic of China

## Abstract

The title complex, [Fe_2_(C_20_H_14_N_2_O_2_)_2_O]·CH_4_O·2H_2_O, is composed of μ-oxido-bridged ferric 2,2′-[*o*-phenylene­bis(nitrilo­methylidyne)]diphenolate (salphen) dimers, one methanol mol­ecule and two H_2_O mol­ecules. Each iron(III) ion, surrounded by two coordinating N and O atoms from the salphen ligand and one bridging O atom, shows a five-coordinate square-pyramidal geometry. One of the two solvent water mol­ecules is disordered over three positions with occupancies of 0.44 (1), 0.37 (1) and 0.19 (1).

## Related literature

For background to μ-oxo-diiron(III) complexes, see: Kurtz *et al.* (1990 ▶); Vincent *et al.* (1990 ▶); Reedijk & Bouwman (1999 ▶); Oyaizu *et al.* (2001 ▶). For related structures, see: Ashmawy & Ujaimi (1991 ▶); Elmali *et al.* (1993 ▶); Oyaizu *et al.* (2001 ▶). 
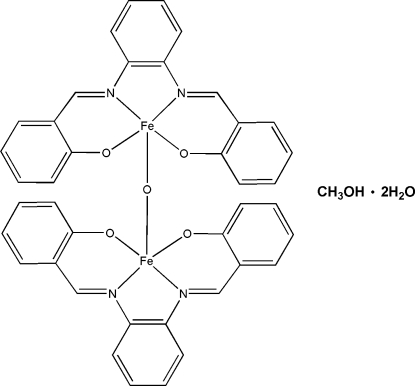


## Experimental

### 

#### Crystal data

[Fe_2_(C_20_H_14_N_2_O_2_)_2_O]·CH_4_O·2H_2_O
*M* *_r_* = 824.44
Triclinic, 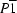

*a* = 13.042 (3) Å
*b* = 13.249 (3) Å
*c* = 13.724 (3) Å
α = 116.60 (3)°
β = 110.50 (3)°
γ = 93.80 (3)°
*V* = 1914.4 (12) Å^3^
*Z* = 2
Mo *K*α radiation
μ = 0.82 mm^−1^
*T* = 298 K
0.22 × 0.20 × 0.20 mm

#### Data collection

Rigaku CCD area-detector diffractometer
Absorption correction: multi-scan (*ABSCOR*; Higashi, 1995 ▶) *T*_min_ = 0.841, *T*_max_ = 0.854
15784 measured reflections
6858 independent reflections
4753 reflections with *I* > 2σ(*I*)
*R*_int_ = 0.040

#### Refinement

*R*[*F* ^2^ > 2σ(*F* ^2^)] = 0.051
*wR*(*F* ^2^) = 0.158
*S* = 1.05
6858 reflections
519 parameters
4 restraints
H-atom parameters constrained
Δρ_max_ = 0.59 e Å^−3^
Δρ_min_ = −0.34 e Å^−3^

### 

Data collection: *CrystalClear* (Rigaku, 2008 ▶); cell refinement: *CrystalClear*; data reduction: *CrystalClear*; program(s) used to solve structure: *SHELXS97* (Sheldrick, 2008 ▶); program(s) used to refine structure: *SHELXL97* (Sheldrick, 2008 ▶); molecular graphics: *DIAMOND* (Brandenburg, 2006 ▶); software used to prepare material for publication: *SHELXTL* (Sheldrick, 2008 ▶).

## Supplementary Material

Crystal structure: contains datablocks I, global. DOI: 10.1107/S1600536810031533/zl2295sup1.cif

Structure factors: contains datablocks I. DOI: 10.1107/S1600536810031533/zl2295Isup2.hkl

Additional supplementary materials:  crystallographic information; 3D view; checkCIF report

## Figures and Tables

**Table 1:** Hydrogen-bond geometry (Å, °)

*D*—H⋯*A*	*D*—H	H⋯*A*	*D*⋯*A*	*D*—H⋯*A*
O6—H6⋯O7^i^	0.82	2.15	2.916 (6)	156
O7—H7*WB*⋯O6	0.99	1.87	2.808 (6)	158
O7—H7*WA*⋯O3	0.96	2.17	3.090 (5)	160
O7—H7*WA*⋯O4	0.96	2.62	3.330 (5)	131

Symmetry code: (i) 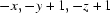.

**Fig. 1.**
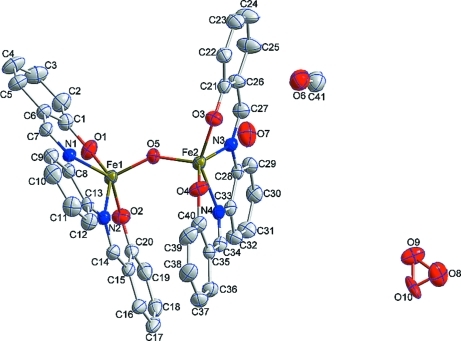
The molecular structure of the title complex, with atom labels and 30% probability displacement ellipsoids; H atoms have been omitted for clarity.

**Fig. 2.**
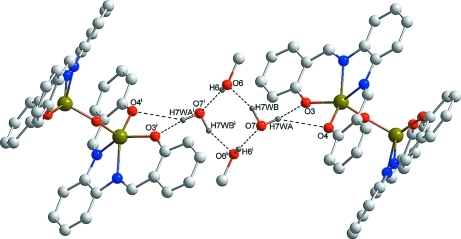
Hydrogen bonding interactions of the title complex; the disordered water molecule is omitted for clarity. Symmetry code: (i) -x, -y+1, -z+1.

## supplementary crystallographic information

### Comment

µ-Oxo-diiron(III) complexes are of considerable interest to chemists and biologists because of their interesting electronic structures and the magnetic interactions between the two iron(III) centers, and the role played by the oxo-bridged dinuclear iron centres in proteins (Kurtz *et al.*, 1990; Vincent *et al.*, 1990; Oyaizu *et al.*, 2001). The Fe—Fe distances and the corresponding the Fe—O—Fe bond lengths and the angles are the most important factors that determine the electronic and magnetic properties of these complexes (Reedijk *et al.*, 1999). It is important to note that the crystal structure of µ-oxo-bridged ferric salphen dimers [salphenH_2_= *N*,*N*'-*o*-phenylenebis(salicylideneimine)] depend strongly on the presence and type of lattice solvent molecules: [{Fe^III^(salphen)}_2_O].CH_2_Cl_2_.C_4_H_10_O (Oyaizu *et al.*, 2001); [{Fe^III^(salphen)}_2_O].DMSO (Ashmawy *et al.*, 1991) and [{Fe^III^(salphen)}_2_O].C_4_H_8_O_2_ (Elmali *et al.*, 1993). By using a different solvent system, we obtained a new methanol dihydrate solvate of the µ-oxo-diiron(III) complex, [{Fe^III^(salphen)}_2_O].CH_3_OH.2H_2_O. Herein, the crystal structure of this solvate is presented.

The title complex is composed of one µ-oxo-diiron(III) unit of [{Fe^III^(salphen)}_2_O], one methanol molecule and two H_2_O molecules (Fig. 1). Each iron(III) atom, surrounded by each two coordinating N and O atoms from the salphen ligand, extends outwards of the mean N_2_O_2_ plane towards the bridging oxygen atom by as much as 0.588 (3) and 0.583 (3) Å for Fe(1) and Fe(2), respectively. The iron atoms thus substantially protrude from the ligand planes and show a typical five-coordinate square-pyramidal geometry. The Fe—O (bridging) bond lengths are 1.786 (3) and 1.784 (3) Å for Fe(1) and Fe(2), respectively. The Fe—O—Fe angle of 146.68 (16)° is almost equal to the value of 146.7 (4)° reported for [{Fe^III^(salphen)}_2_O].DMSO (Ashmawy *et al.*, 1991), and is bigger than the values of 141 (1)° and 145.0 (3)° reported for [{Fe^III^(salphen)}_2_O].CH_2_Cl_2_.C_4_H_10_O (Oyaizu *et al.*, 2001) and [{Fe^III^(salphen)}_2_O].C_4_H_8_O_2_ (Elmali *et al.*, 1993), respectively. The Fe···Fe distance of 3.420 (3) Å is consistent with the values (3.35–3.55 Å) reported for µ-oxo-diiron(III) complexes with macrocyclic ligands (Oyaizu *et al.*, 2001). One of the two interstitial water molecules in the structure was found to be svererly disordered and has been refined as disordered over three positions with occupancies of 43.9 (4)%, 37 (1)% and 19 (1)% for O8, O9 and O10, respectively. Hydrogen atoms for the disordered water molecule could not be located and were omitted from the refinement.

There are some hydrogen-bonding interactions between methanol and water molecules, and between the water molecules and the salphen ligand. These hydrogen bonding interactions lead to a group of four oxygen atoms - two water and two methanol molecules - that are arranged around a crystallographic inversion center in a quadratic square pattern. The water molecules of the unit form additional bifurcated hydrogen bonds twoards the two oxygen atoms (O3, O4) of a salphen ligand of adjacent [{Fe^III^(salphen)}_2_O] complexes thus binding the complexes together by H-bonds via the square H_2_O/MeOH units (Table 1, Fig. 2). The oxygen atoms of the other salphen ligand of the complex (O1, O2) show signs of hydrogen bonding interactions with the disordered water molecule.

### Experimental

Red prismatic crystals of the title complex were obtained by slow evaporation of a MeOH and H_2_O (V/V = 1:1, 10 mL) mixture of {Fe(salphen)(C_2_H_5_OH)_2_}Cl (0.1 mmol) in the dark at room temperature. The resulting crystals were collected, washed with H_2_O and MeOH, respectively, and dried in air. Melting point = 446.6 K. IR (KBr, cm^-1^): 3416(s), 2958(m), 2921(m), 2115(w), 1605(s), 1581(s), 1532(s), 1462(s), 1446(m), 1381(m), 1323(m), 1193(m), 1158(m), 1050(m), 745(m), 540(m).

### Refinement

All non-H atoms were refined anisotropically. The (C)H atoms of the salphenH_2_ ligand were placed in calculated positions (C - H = 0.93 Å) and refined using a riding model, with *U*_iso_(H) = 1.2*U*_eq_(C). The (C)H atoms of the methanol molecule were placed geometrically (C - H = 0.96 Å) and refined as riding, with *U*_iso_(H) = 1.5*U*_eq_(C). The (O)H atoms of the methanol molecule was placed geometrically (O - H = 0.82 Å) with *U*_iso_(H) = 1.5*U*_eq_(O). The (O)H atoms of water molecule (O7) were located in a difference Fourier map and refined as riding with *U*_iso_(H) = 1.2*U*_eq_(O). The other water molecule in the structure was found to be svererly disordered and has been refined as disordered over three positions with occupancies of 43.9 (4)%, 37 (1)% and 19 (1)% for O8, O9 and O10, respectively, summing up to 100%. Hydrogen atoms for the disordered water molecule could not be loctated and were omitted from the refinement.

### Crystal data

**Table:**

[Fe_2_(C_20_H_14_N_2_O_2_)_2_O]·CH_4_O·2H_2_O	*Z* = 2
*M**_r_* = 824.44	*F*(000) = 848
Triclinic, *P*1	*D*_x_ = 1.427 Mg m^−^^3^
Hall symbol: -P 1	Melting point: 446.6 K
*a* = 13.042 (3) Å	Mo *K*α radiation, λ = 0.71073 Å
*b* = 13.249 (3) Å	Cell parameters from 7899 reflections
*c* = 13.724 (3) Å	θ = 2.7–28.9°
α = 116.60 (3)°	µ = 0.82 mm^−^^1^
β = 110.50 (3)°	*T* = 298 K
γ = 93.80 (3)°	Prism, red
*V* = 1914.4 (12) Å^3^	0.22 × 0.20 × 0.20 mm

### Data collection

**Table:**

Rigaku Model? CCD area-detector diffractometer	6858 independent reflections
Radiation source: fine-focus sealed tube	4753 reflections with *I* > 2σ(*I*)
graphite	*R*_int_ = 0.040
Detector resolution: 14.63 pixels mm^-1^	θ_max_ = 25.3°, θ_min_ = 3.0°
phi and ω scans	*h* = −13→15
Absorption correction: multi-scan (*ABSCOR*; Higashi, 1995)	*k* = −15→15
*T*_min_ = 0.841, *T*_max_ = 0.854	*l* = −13→16
15784 measured reflections	

### Refinement

**Table:**

Refinement on *F*^2^	Primary atom site location: structure-invariant direct methods
Least-squares matrix: full	Secondary atom site location: difference Fourier map
*R*[*F*^2^ > 2σ(*F*^2^)] = 0.051	Hydrogen site location: inferred from neighbouring sites
*wR*(*F*^2^) = 0.158	H-atom parameters constrained
*S* = 1.05	*w* = 1/[σ^2^(*F*_o_^2^) + (0.0832*P*)^2^] where *P* = (*F*_o_^2^ + 2*F*_c_^2^)/3
6858 reflections	(Δ/σ)_max_ < 0.001
519 parameters	Δρ_max_ = 0.59 e Å^−^^3^
4 restraints	Δρ_min_ = −0.34 e Å^−^^3^

### Special details

**Table:**

Geometry. All esds (except the esd in the dihedral angle between two l.s. planes) are estimated using the full covariance matrix. The cell esds are taken into account individually in the estimation of esds in distances, angles and torsion angles; correlations between esds in cell parameters are only used when they are defined by crystal symmetry. An approximate (isotropic) treatment of cell esds is used for estimating esds involving l.s. planes.
Refinement. Refinement of F^2^ against ALL reflections. The weighted R-factor wR and goodness of fit S are based on F^2^, conventional R-factors R are based on F, with F set to zero for negative F^2^. The threshold expression of F^2^ > 2sigma(F^2^) is used only for calculating R-factors(gt) etc. and is not relevant to the choice of reflections for refinement. R-factors based on F^2^ are statistically about twice as large as those based on F, and R- factors based on ALL data will be even larger.

### Fractional atomic coordinates and isotropic or equivalent isotropic displacement parameters (Å^2^)

**Table:**

	*x*	*y*	*z*	*U*_iso_*/*U*_eq_	Occ. (<1)
Fe1	0.29364 (4)	0.63148 (4)	0.11270 (5)	0.04383 (19)	
Fe2	0.31765 (4)	0.64507 (4)	0.37482 (4)	0.04180 (19)	
O1	0.2687 (3)	0.7573 (2)	0.0824 (3)	0.0654 (8)	
O2	0.4527 (2)	0.6843 (2)	0.1582 (3)	0.0634 (8)	
O3	0.1917 (2)	0.6451 (2)	0.4169 (2)	0.0561 (7)	
O4	0.3020 (2)	0.4836 (2)	0.3307 (3)	0.0613 (8)	
O5	0.2735 (2)	0.6584 (2)	0.2435 (2)	0.0512 (7)	
O6	0.1044 (3)	0.5981 (4)	0.6541 (4)	0.1005 (12)	
H6	0.0414	0.6072	0.6256	0.151*	
O7	0.1409 (4)	0.4394 (4)	0.4574 (3)	0.1024 (12)	
O8	0.4974 (9)	0.0920 (8)	0.9275 (10)	0.103 (3)	0.439 (4)
O9	0.4522 (8)	0.1400 (10)	0.8664 (11)	0.077 (5)	0.367 (14)
O10	0.5116 (18)	0.0771 (17)	0.8511 (15)	0.068 (10)	0.194 (14)
N1	0.1355 (2)	0.5283 (3)	−0.0380 (3)	0.0431 (7)	
N2	0.3115 (3)	0.4595 (3)	0.0466 (3)	0.0433 (7)	
N3	0.3882 (2)	0.8219 (3)	0.5153 (3)	0.0432 (7)	
N4	0.4954 (2)	0.6660 (2)	0.4389 (3)	0.0408 (7)	
C1	0.1774 (4)	0.7752 (4)	0.0167 (4)	0.0558 (10)	
C2	0.1821 (4)	0.8892 (4)	0.0354 (5)	0.0755 (14)	
H2	0.2464	0.9502	0.0955	0.091*	
C3	0.0925 (5)	0.9108 (5)	−0.0347 (6)	0.0900 (17)	
H3	0.0971	0.9865	−0.0215	0.108*	
C4	−0.0050 (5)	0.8223 (5)	−0.1247 (6)	0.0900 (17)	
H4	−0.0641	0.8379	−0.1732	0.108*	
C5	−0.0128 (4)	0.7132 (4)	−0.1409 (5)	0.0703 (13)	
H5	−0.0794	0.6545	−0.1992	0.084*	
C6	0.0777 (3)	0.6856 (4)	−0.0714 (4)	0.0529 (10)	
C7	0.0635 (3)	0.5673 (4)	−0.0968 (3)	0.0502 (10)	
H7	−0.0032	0.5130	−0.1613	0.060*	
C8	0.1129 (3)	0.4083 (3)	−0.0722 (3)	0.0434 (9)	
C9	0.0074 (3)	0.3275 (4)	−0.1464 (4)	0.0544 (10)	
H9	−0.0557	0.3503	−0.1787	0.065*	
C10	−0.0034 (4)	0.2133 (4)	−0.1720 (4)	0.0692 (13)	
H10	−0.0740	0.1590	−0.2226	0.083*	
C11	0.0890 (4)	0.1786 (4)	−0.1238 (4)	0.0688 (13)	
H11	0.0800	0.1016	−0.1404	0.083*	
C12	0.1933 (4)	0.2558 (4)	−0.0520 (4)	0.0597 (11)	
H12	0.2553	0.2313	−0.0203	0.072*	
C13	0.2079 (3)	0.3717 (3)	−0.0258 (3)	0.0426 (9)	
C14	0.4083 (3)	0.4325 (4)	0.0679 (4)	0.0511 (10)	
H14	0.4054	0.3534	0.0367	0.061*	
C15	0.5183 (3)	0.5133 (4)	0.1349 (3)	0.0508 (10)	
C16	0.6140 (4)	0.4682 (5)	0.1558 (4)	0.0675 (13)	
H16	0.6028	0.3884	0.1278	0.081*	
C17	0.7222 (4)	0.5385 (6)	0.2158 (5)	0.0794 (16)	
H17	0.7839	0.5074	0.2313	0.095*	
C18	0.7388 (4)	0.6556 (6)	0.2532 (4)	0.0799 (16)	
H18	0.8125	0.7031	0.2921	0.096*	
C19	0.6498 (4)	0.7042 (5)	0.2346 (4)	0.0702 (13)	
H19	0.6635	0.7838	0.2604	0.084*	
C20	0.5363 (3)	0.6337 (4)	0.1761 (4)	0.0548 (11)	
C21	0.1505 (3)	0.7323 (4)	0.4700 (4)	0.0517 (10)	
C22	0.0402 (4)	0.7060 (4)	0.4609 (4)	0.0624 (12)	
H22	−0.0027	0.6284	0.4162	0.075*	
C23	−0.0049 (4)	0.7924 (5)	0.5166 (5)	0.0818 (16)	
H23	−0.0777	0.7725	0.5099	0.098*	
C24	0.0557 (5)	0.9092 (5)	0.5830 (6)	0.101 (2)	
H24	0.0229	0.9676	0.6177	0.121*	
C25	0.1636 (4)	0.9372 (4)	0.5965 (5)	0.0924 (18)	
H25	0.2060	1.0151	0.6449	0.111*	
C26	0.2139 (4)	0.8500 (4)	0.5382 (4)	0.0590 (11)	
C27	0.3295 (4)	0.8878 (4)	0.5621 (4)	0.0578 (11)	
H27	0.3662	0.9668	0.6161	0.069*	
C28	0.5039 (3)	0.8682 (3)	0.5481 (3)	0.0424 (9)	
C29	0.5628 (3)	0.9868 (3)	0.6164 (4)	0.0525 (10)	
H29	0.5259	1.0428	0.6464	0.063*	
C30	0.6742 (4)	1.0215 (4)	0.6398 (4)	0.0614 (11)	
H30	0.7124	1.1007	0.6847	0.074*	
C31	0.7297 (4)	0.9379 (4)	0.5960 (4)	0.0645 (12)	
H31	0.8055	0.9614	0.6125	0.077*	
C32	0.6732 (3)	0.8196 (4)	0.5278 (4)	0.0558 (11)	
H32	0.7105	0.7644	0.4972	0.067*	
C33	0.5609 (3)	0.7839 (3)	0.5052 (3)	0.0427 (9)	
C34	0.5450 (3)	0.5817 (3)	0.4215 (3)	0.0449 (9)	
H34	0.6239	0.6026	0.4540	0.054*	
C35	0.4890 (3)	0.4598 (3)	0.3569 (3)	0.0432 (9)	
C36	0.5582 (4)	0.3811 (3)	0.3384 (4)	0.0558 (10)	
H36	0.6362	0.4106	0.3696	0.067*	
C37	0.5117 (4)	0.2625 (4)	0.2752 (4)	0.0658 (12)	
H37	0.5577	0.2118	0.2625	0.079*	
C38	0.3965 (4)	0.2190 (4)	0.2305 (4)	0.0693 (13)	
H38	0.3649	0.1384	0.1866	0.083*	
C39	0.3272 (4)	0.2930 (4)	0.2499 (4)	0.0691 (13)	
H39	0.2498	0.2615	0.2209	0.083*	
C40	0.3718 (3)	0.4161 (3)	0.3133 (4)	0.0503 (10)	
C41	0.1861 (5)	0.7092 (5)	0.7313 (5)	0.0967 (18)	
H41A	0.1500	0.7682	0.7646	0.145*	
H41B	0.2180	0.7274	0.6859	0.145*	
H41C	0.2453	0.7068	0.7952	0.145*	
H7WB	0.1494	0.5026	0.5366	0.116*	
H7WA	0.1725	0.4961	0.4429	0.116*	

### Atomic displacement parameters (Å^2^)

**Table:**

	*U*^11^	*U*^22^	*U*^33^	*U*^12^	*U*^13^	*U*^23^
Fe1	0.0386 (3)	0.0414 (3)	0.0389 (3)	0.0049 (3)	0.0090 (2)	0.0168 (3)
Fe2	0.0351 (3)	0.0388 (3)	0.0406 (3)	0.0037 (2)	0.0097 (2)	0.0171 (3)
O1	0.0621 (19)	0.0489 (16)	0.0628 (19)	0.0002 (14)	0.0035 (15)	0.0300 (15)
O2	0.0445 (16)	0.0534 (17)	0.071 (2)	0.0017 (14)	0.0187 (15)	0.0212 (15)
O3	0.0485 (16)	0.0500 (16)	0.0631 (18)	0.0052 (14)	0.0257 (14)	0.0227 (14)
O4	0.0406 (15)	0.0438 (15)	0.086 (2)	0.0047 (13)	0.0159 (15)	0.0312 (15)
O5	0.0489 (15)	0.0534 (15)	0.0409 (15)	0.0117 (13)	0.0140 (12)	0.0198 (13)
O6	0.082 (3)	0.105 (3)	0.094 (3)	0.013 (2)	0.025 (2)	0.044 (2)
O7	0.104 (3)	0.107 (3)	0.084 (3)	−0.006 (2)	0.033 (2)	0.048 (2)
O8	0.151 (9)	0.083 (6)	0.102 (7)	0.030 (6)	0.061 (7)	0.064 (6)
O9	0.056 (6)	0.060 (6)	0.130 (9)	0.008 (5)	0.036 (6)	0.063 (6)
O10	0.069 (14)	0.057 (12)	0.036 (10)	−0.037 (10)	0.033 (8)	−0.009 (7)
N1	0.0367 (17)	0.0464 (18)	0.0414 (18)	0.0088 (15)	0.0140 (14)	0.0206 (15)
N2	0.0392 (17)	0.0455 (17)	0.0414 (18)	0.0088 (15)	0.0137 (14)	0.0218 (15)
N3	0.0376 (17)	0.0428 (17)	0.0430 (18)	0.0070 (15)	0.0128 (15)	0.0204 (14)
N4	0.0364 (16)	0.0388 (16)	0.0364 (17)	0.0026 (14)	0.0103 (14)	0.0154 (14)
C1	0.064 (3)	0.051 (2)	0.057 (3)	0.015 (2)	0.025 (2)	0.032 (2)
C2	0.077 (3)	0.064 (3)	0.087 (4)	0.015 (3)	0.026 (3)	0.046 (3)
C3	0.092 (4)	0.077 (4)	0.136 (5)	0.038 (3)	0.053 (4)	0.075 (4)
C4	0.072 (4)	0.097 (4)	0.128 (5)	0.038 (3)	0.032 (4)	0.082 (4)
C5	0.053 (3)	0.078 (3)	0.083 (3)	0.026 (3)	0.021 (2)	0.047 (3)
C6	0.054 (3)	0.056 (2)	0.054 (3)	0.020 (2)	0.023 (2)	0.031 (2)
C7	0.037 (2)	0.058 (2)	0.042 (2)	0.0086 (19)	0.0101 (17)	0.0205 (19)
C8	0.039 (2)	0.040 (2)	0.039 (2)	0.0032 (17)	0.0140 (17)	0.0138 (17)
C9	0.040 (2)	0.056 (3)	0.050 (2)	0.001 (2)	0.0145 (19)	0.019 (2)
C10	0.057 (3)	0.052 (3)	0.063 (3)	−0.014 (2)	0.015 (2)	0.012 (2)
C11	0.075 (3)	0.043 (2)	0.072 (3)	0.000 (2)	0.024 (3)	0.024 (2)
C12	0.065 (3)	0.049 (2)	0.060 (3)	0.012 (2)	0.021 (2)	0.029 (2)
C13	0.044 (2)	0.040 (2)	0.035 (2)	0.0047 (18)	0.0138 (17)	0.0149 (17)
C14	0.054 (3)	0.057 (2)	0.051 (2)	0.020 (2)	0.022 (2)	0.033 (2)
C15	0.038 (2)	0.073 (3)	0.043 (2)	0.013 (2)	0.0155 (19)	0.032 (2)
C16	0.057 (3)	0.105 (4)	0.067 (3)	0.036 (3)	0.033 (2)	0.057 (3)
C17	0.043 (3)	0.146 (5)	0.062 (3)	0.033 (3)	0.022 (2)	0.061 (4)
C18	0.038 (3)	0.131 (5)	0.049 (3)	0.004 (3)	0.013 (2)	0.035 (3)
C19	0.047 (3)	0.083 (3)	0.051 (3)	−0.005 (3)	0.016 (2)	0.019 (2)
C20	0.038 (2)	0.076 (3)	0.043 (2)	0.007 (2)	0.0164 (19)	0.026 (2)
C21	0.040 (2)	0.062 (3)	0.045 (2)	0.007 (2)	0.0121 (19)	0.026 (2)
C22	0.044 (2)	0.075 (3)	0.056 (3)	0.009 (2)	0.018 (2)	0.027 (2)
C23	0.042 (3)	0.102 (4)	0.072 (3)	0.013 (3)	0.020 (3)	0.024 (3)
C24	0.058 (3)	0.092 (4)	0.119 (5)	0.027 (3)	0.044 (3)	0.022 (4)
C25	0.067 (3)	0.059 (3)	0.113 (5)	0.012 (3)	0.040 (3)	0.012 (3)
C26	0.046 (2)	0.055 (3)	0.067 (3)	0.013 (2)	0.024 (2)	0.023 (2)
C27	0.051 (2)	0.043 (2)	0.059 (3)	0.006 (2)	0.018 (2)	0.015 (2)
C28	0.040 (2)	0.042 (2)	0.035 (2)	0.0020 (17)	0.0100 (17)	0.0169 (17)
C29	0.054 (3)	0.039 (2)	0.051 (2)	0.0057 (19)	0.019 (2)	0.0158 (18)
C30	0.052 (3)	0.047 (2)	0.056 (3)	−0.007 (2)	0.014 (2)	0.013 (2)
C31	0.042 (2)	0.058 (3)	0.070 (3)	−0.006 (2)	0.020 (2)	0.019 (2)
C32	0.042 (2)	0.048 (2)	0.057 (3)	0.0008 (19)	0.018 (2)	0.014 (2)
C33	0.038 (2)	0.041 (2)	0.036 (2)	−0.0014 (17)	0.0097 (17)	0.0158 (17)
C34	0.039 (2)	0.049 (2)	0.043 (2)	0.0033 (18)	0.0144 (18)	0.0241 (18)
C35	0.048 (2)	0.041 (2)	0.038 (2)	0.0071 (18)	0.0160 (18)	0.0213 (17)
C36	0.060 (3)	0.049 (2)	0.059 (3)	0.017 (2)	0.024 (2)	0.029 (2)
C37	0.077 (3)	0.058 (3)	0.070 (3)	0.030 (3)	0.034 (3)	0.035 (2)
C38	0.076 (3)	0.040 (2)	0.070 (3)	0.011 (2)	0.014 (3)	0.024 (2)
C39	0.052 (3)	0.050 (3)	0.080 (3)	0.001 (2)	0.005 (2)	0.031 (2)
C40	0.048 (2)	0.041 (2)	0.052 (2)	0.0055 (19)	0.0114 (19)	0.0236 (19)
C41	0.082 (4)	0.111 (5)	0.085 (4)	0.017 (4)	0.027 (3)	0.047 (4)

### Geometric parameters (Å, °)

**Table:**

Fe1—O5	1.786 (3)	C12—H12	0.9300
Fe1—O1	1.912 (3)	C14—C15	1.429 (5)
Fe1—O2	1.921 (3)	C14—H14	0.9300
Fe1—N2	2.106 (3)	C15—C20	1.404 (6)
Fe1—N1	2.123 (3)	C15—C16	1.413 (5)
Fe2—O5	1.784 (3)	C16—C17	1.365 (7)
Fe2—O4	1.919 (3)	C16—H16	0.9300
Fe2—O3	1.922 (3)	C17—C18	1.372 (7)
Fe2—N3	2.116 (3)	C17—H17	0.9300
Fe2—N4	2.119 (3)	C18—C19	1.366 (7)
O1—C1	1.329 (5)	C18—H18	0.9300
O2—C20	1.325 (5)	C19—C20	1.421 (6)
O3—C21	1.327 (5)	C19—H19	0.9300
O4—C40	1.325 (5)	C21—C26	1.403 (6)
O6—C41	1.424 (6)	C21—C22	1.405 (6)
O6—H6	0.8200	C22—C23	1.364 (6)
O7—H7WB	0.9910	C22—H22	0.9300
O7—H7WA	0.9611	C23—C24	1.386 (7)
O8—O10	1.063 (14)	C23—H23	0.9300
O8—O9	1.280 (12)	C24—C25	1.356 (7)
O9—O10	1.18 (2)	C24—H24	0.9300
N1—C7	1.307 (5)	C25—C26	1.430 (6)
N1—C8	1.416 (5)	C25—H25	0.9300
N2—C14	1.304 (5)	C26—C27	1.428 (6)
N2—C13	1.409 (5)	C27—H27	0.9300
N3—C27	1.301 (5)	C28—C29	1.398 (5)
N3—C28	1.418 (5)	C28—C33	1.405 (5)
N4—C34	1.300 (5)	C29—C30	1.371 (6)
N4—C33	1.416 (4)	C29—H29	0.9300
C1—C6	1.403 (6)	C30—C31	1.388 (6)
C1—C2	1.407 (6)	C30—H30	0.9300
C2—C3	1.370 (7)	C31—C32	1.387 (6)
C2—H2	0.9300	C31—H31	0.9300
C3—C4	1.386 (8)	C32—C33	1.387 (5)
C3—H3	0.9300	C32—H32	0.9300
C4—C5	1.351 (7)	C34—C35	1.427 (5)
C4—H4	0.9300	C34—H34	0.9300
C5—C6	1.421 (6)	C35—C40	1.400 (5)
C5—H5	0.9300	C35—C36	1.418 (5)
C6—C7	1.428 (6)	C36—C37	1.369 (6)
C7—H7	0.9300	C36—H36	0.9300
C8—C9	1.387 (5)	C37—C38	1.374 (6)
C8—C13	1.408 (5)	C37—H37	0.9300
C9—C10	1.376 (6)	C38—C39	1.379 (6)
C9—H9	0.9300	C38—H38	0.9300
C10—C11	1.374 (7)	C39—C40	1.416 (6)
C10—H10	0.9300	C39—H39	0.9300
C11—C12	1.357 (6)	C41—H41A	0.9600
C11—H11	0.9300	C41—H41B	0.9600
C12—C13	1.393 (5)	C41—H41C	0.9600
			
O5—Fe1—O1	109.47 (13)	C20—C15—C16	118.4 (4)
O5—Fe1—O2	109.06 (13)	C20—C15—C14	123.6 (4)
O1—Fe1—O2	89.13 (13)	C16—C15—C14	117.9 (4)
O5—Fe1—N2	102.31 (12)	C17—C16—C15	121.9 (5)
O1—Fe1—N2	147.27 (13)	C17—C16—H16	119.0
O2—Fe1—N2	87.79 (13)	C15—C16—H16	119.0
O5—Fe1—N1	106.82 (12)	C16—C17—C18	119.1 (5)
O1—Fe1—N1	87.13 (12)	C16—C17—H17	120.4
O2—Fe1—N1	143.05 (12)	C18—C17—H17	120.4
N2—Fe1—N1	76.19 (12)	C19—C18—C17	121.7 (5)
O5—Fe2—O4	110.14 (13)	C19—C18—H18	119.1
O5—Fe2—O3	107.68 (12)	C17—C18—H18	119.1
O4—Fe2—O3	90.01 (12)	C18—C19—C20	120.3 (5)
O5—Fe2—N3	102.33 (12)	C18—C19—H19	119.9
O4—Fe2—N3	146.57 (13)	C20—C19—H19	119.9
O3—Fe2—N3	87.54 (12)	O2—C20—C15	123.3 (4)
O5—Fe2—N4	106.81 (12)	O2—C20—C19	118.2 (4)
O4—Fe2—N4	87.08 (12)	C15—C20—C19	118.5 (4)
O3—Fe2—N4	144.17 (12)	O3—C21—C26	122.6 (3)
N3—Fe2—N4	75.93 (12)	O3—C21—C22	118.8 (4)
C1—O1—Fe1	133.1 (3)	C26—C21—C22	118.5 (4)
C20—O2—Fe1	131.4 (3)	C23—C22—C21	121.0 (5)
C21—O3—Fe2	130.6 (3)	C23—C22—H22	119.5
C40—O4—Fe2	131.8 (3)	C21—C22—H22	119.5
Fe2—O5—Fe1	146.68 (16)	C22—C23—C24	121.4 (5)
C41—O6—H6	109.5	C22—C23—H23	119.3
H7WB—O7—H7WA	90.6	C24—C23—H23	119.3
O10—O8—O9	59.4 (14)	C25—C24—C23	119.0 (5)
O10—O9—O8	51.1 (9)	C25—C24—H24	120.5
O8—O10—O9	69.5 (12)	C23—C24—H24	120.5
C7—N1—C8	121.2 (3)	C24—C25—C26	121.7 (5)
C7—N1—Fe1	125.4 (3)	C24—C25—H25	119.2
C8—N1—Fe1	113.4 (2)	C26—C25—H25	119.2
C14—N2—C13	120.9 (3)	C21—C26—C27	124.1 (4)
C14—N2—Fe1	124.9 (3)	C21—C26—C25	118.3 (4)
C13—N2—Fe1	114.2 (2)	C27—C26—C25	117.3 (4)
C27—N3—C28	121.3 (3)	N3—C27—C26	126.0 (4)
C27—N3—Fe2	123.7 (3)	N3—C27—H27	117.0
C28—N3—Fe2	114.8 (2)	C26—C27—H27	117.0
C34—N4—C33	120.2 (3)	C29—C28—C33	119.3 (3)
C34—N4—Fe2	125.6 (2)	C29—C28—N3	126.0 (3)
C33—N4—Fe2	114.2 (2)	C33—C28—N3	114.7 (3)
O1—C1—C6	123.0 (4)	C30—C29—C28	120.9 (4)
O1—C1—C2	118.1 (4)	C30—C29—H29	119.6
C6—C1—C2	118.8 (4)	C28—C29—H29	119.6
C3—C2—C1	120.3 (5)	C29—C30—C31	119.6 (4)
C3—C2—H2	119.9	C29—C30—H30	120.2
C1—C2—H2	119.9	C31—C30—H30	120.2
C2—C3—C4	121.5 (5)	C32—C31—C30	120.7 (4)
C2—C3—H3	119.2	C32—C31—H31	119.7
C4—C3—H3	119.2	C30—C31—H31	119.7
C5—C4—C3	119.0 (5)	C33—C32—C31	119.9 (4)
C5—C4—H4	120.5	C33—C32—H32	120.0
C3—C4—H4	120.5	C31—C32—H32	120.0
C4—C5—C6	121.9 (5)	C32—C33—C28	119.6 (3)
C4—C5—H5	119.1	C32—C33—N4	125.0 (3)
C6—C5—H5	119.1	C28—C33—N4	115.4 (3)
C1—C6—C5	118.4 (4)	N4—C34—C35	125.7 (3)
C1—C6—C7	123.4 (4)	N4—C34—H34	117.2
C5—C6—C7	118.2 (4)	C35—C34—H34	117.2
N1—C7—C6	126.0 (4)	C40—C35—C36	119.8 (4)
N1—C7—H7	117.0	C40—C35—C34	123.4 (3)
C6—C7—H7	117.0	C36—C35—C34	116.8 (3)
C9—C8—C13	119.3 (4)	C37—C36—C35	121.0 (4)
C9—C8—N1	125.4 (4)	C37—C36—H36	119.5
C13—C8—N1	115.2 (3)	C35—C36—H36	119.5
C10—C9—C8	119.7 (4)	C36—C37—C38	119.6 (4)
C10—C9—H9	120.1	C36—C37—H37	120.2
C8—C9—H9	120.1	C38—C37—H37	120.2
C11—C10—C9	120.7 (4)	C37—C38—C39	121.0 (4)
C11—C10—H10	119.6	C37—C38—H38	119.5
C9—C10—H10	119.6	C39—C38—H38	119.5
C12—C11—C10	120.6 (4)	C38—C39—C40	121.1 (4)
C12—C11—H11	119.7	C38—C39—H39	119.4
C10—C11—H11	119.7	C40—C39—H39	119.5
C11—C12—C13	120.3 (4)	O4—C40—C35	123.5 (3)
C11—C12—H12	119.9	O4—C40—C39	118.9 (4)
C13—C12—H12	119.8	C35—C40—C39	117.6 (4)
C12—C13—C8	119.3 (4)	O6—C41—H41A	109.5
C12—C13—N2	125.6 (4)	O6—C41—H41B	109.5
C8—C13—N2	115.1 (3)	H41A—C41—H41B	109.5
N2—C14—C15	126.0 (4)	O6—C41—H41C	109.5
N2—C14—H14	117.0	H41A—C41—H41C	109.5
C15—C14—H14	117.0	H41B—C41—H41C	109.5

### Hydrogen-bond geometry (Å, °)

**Table:**

*D*—H···*A*	*D*—H	H···*A*	*D*···*A*	*D*—H···*A*
O6—H6···O7^i^	0.82	2.15	2.916 (6)	156.
O7—H7WB···O6	0.99	1.87	2.808 (6)	158.
O7—H7WA···O3	0.96	2.17	3.090 (5)	160.
O7—H7WA···O4	0.96	2.62	3.330 (5)	131.

Symmetry codes: (i) −*x*, −*y*+1, −*z*+1.
